# Supplementary material for: Machine learning reveals limited contribution of trans-only encoded variants to the HLA-DQ immunopeptidome
Source: Commun Biol. 2023 Apr 21;6:442. doi: 10.1038/s42003-023-04749-7 (PMC10121683; doi:10.1038/s42003-023-04749-7)
Supplement: Supplementary file 1 — Supplementary Material [file 42003_2023_4749_MOESM1_ESM.pdf]

Supplementary figures

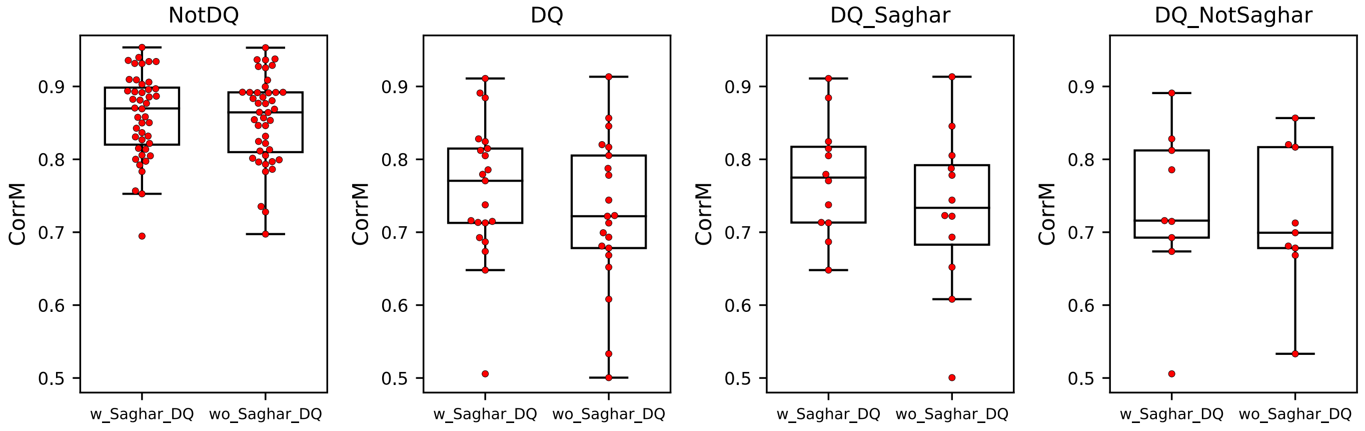

Supplementary figure 1: Mean consistency values for HLA molecule deconvolutions shared between multiple data sets for the two models. Each point reflects an HLA class II molecule, and values shown are the mean correlations over the different data set comparisons. The different subgroups of HLA molecules are non-DQ molecules (NotDQ, n=44), all DQ molecules (DQ, n=21), DQ molecules present in the novel data (DQ_Saghar, n=12), and DQ molecules not present in the novel data (DQ_NotSaghar, n=9). Each boxplot shows the median inside the IQR between the upper and lower quartiles, with whiskers extending to at most 1.5 times the IQR.

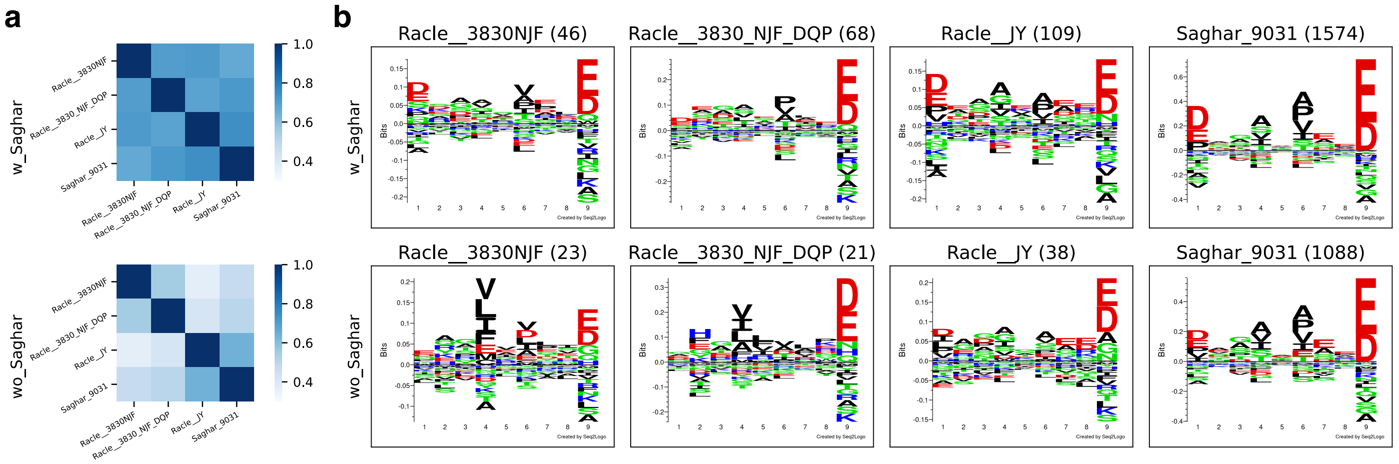

Supplementary figure 2: Consistency analysis for HLA-DQA1*03:01-DQB1*03:02. Here, only a subset of the cell lines used in the consistency analysis are included. **a** Correlation matrices showing the consistency values per cell line pair in the methods trained with (w_Saghar) and without (wo_Saghar) the novel data. **b** Sequence logos constructed from the peptide sets used in the consistency correlation analysis, with a minimum threshold of 20 peptides required for a given logo. The rows correspond to the methods including (w_Saghar) and not including (wo_Saghar) the novel data. The peptide count for each logo is shown in parenthesis in the logo title.

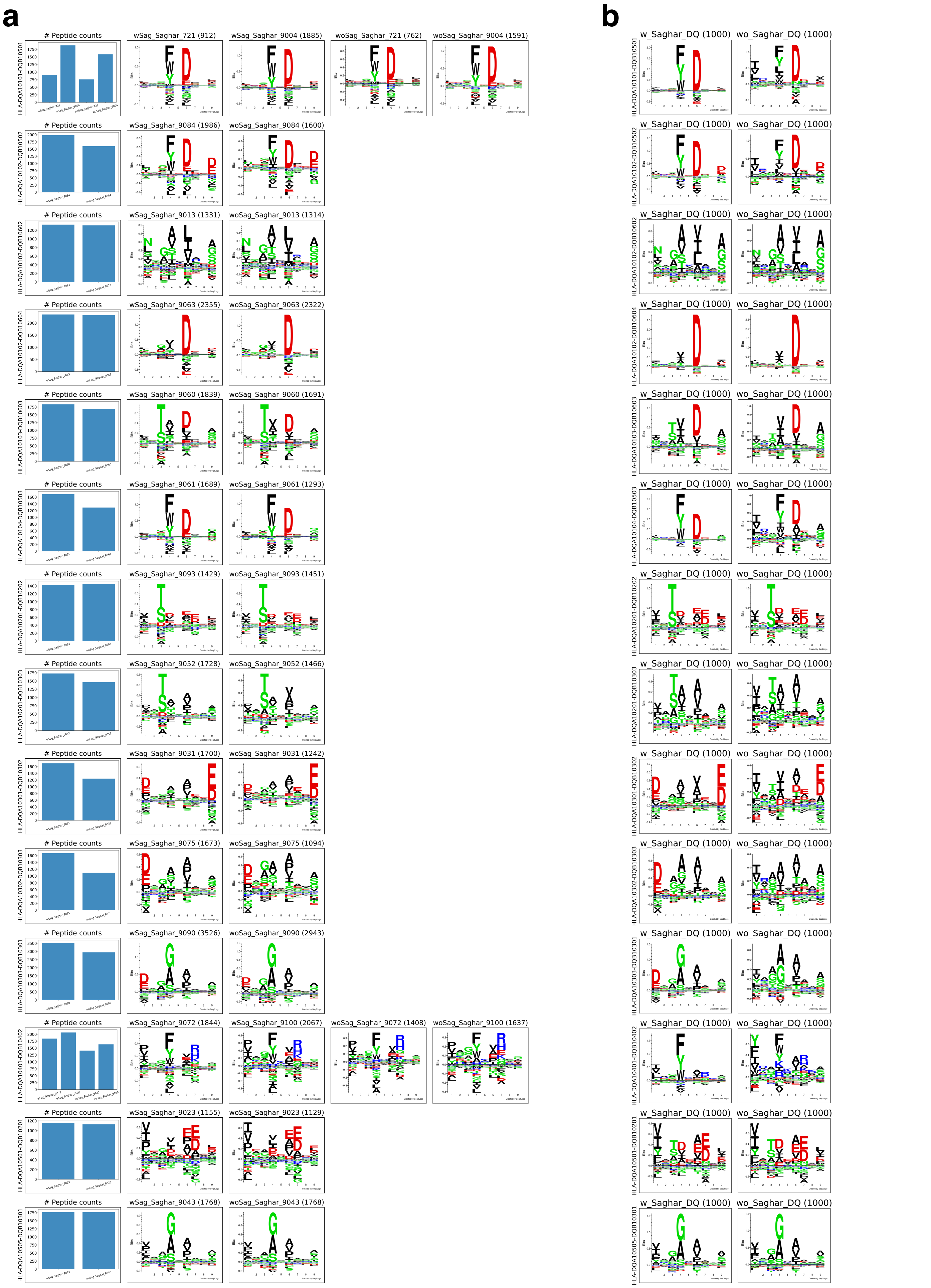

Supplementary figure 3: Identified binding motifs for the 14 DQ molecules from the novel data in the two methods. **a** Motif deconvolution of the novel datasets in the models trained with (wSag) and without (woSag) the novel data (the two rows with 4 logos correspond to the two HLA-DQ molecules shared between two cell lines). **b** Motifs predicted for the HLA-DQ molecules by the two models. Predicted motifs are generated from the top 1% of 100,000 random natural 13-17 mer peptides.

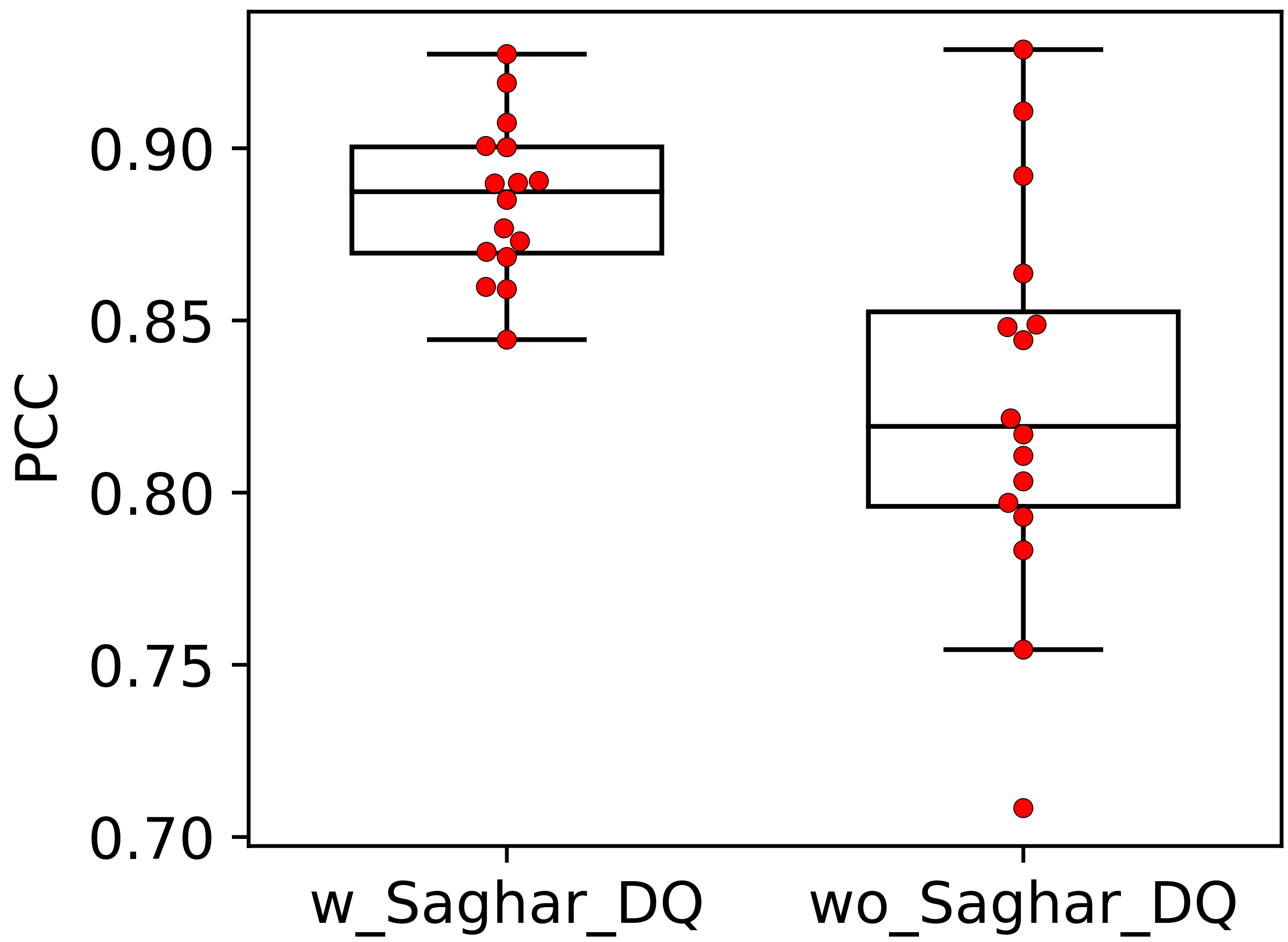

Supplementary figure 4: Correlations between deconvoluted and predicted sequence logos in the methods with (w_Saghar) and without (wo_Saghar) the novel data. For each cell line in the novel data, the PCC value between the DQ motif deconvolution PSSM and predicted PSSM for the corresponding molecule was calculated for each of the methods with and without the novel data. Each boxplot (n=16 logo pairs in both cases) shows the median inside the IQR between the upper and lower quartiles, with whiskers extending to at most 1.5 times the IQR.

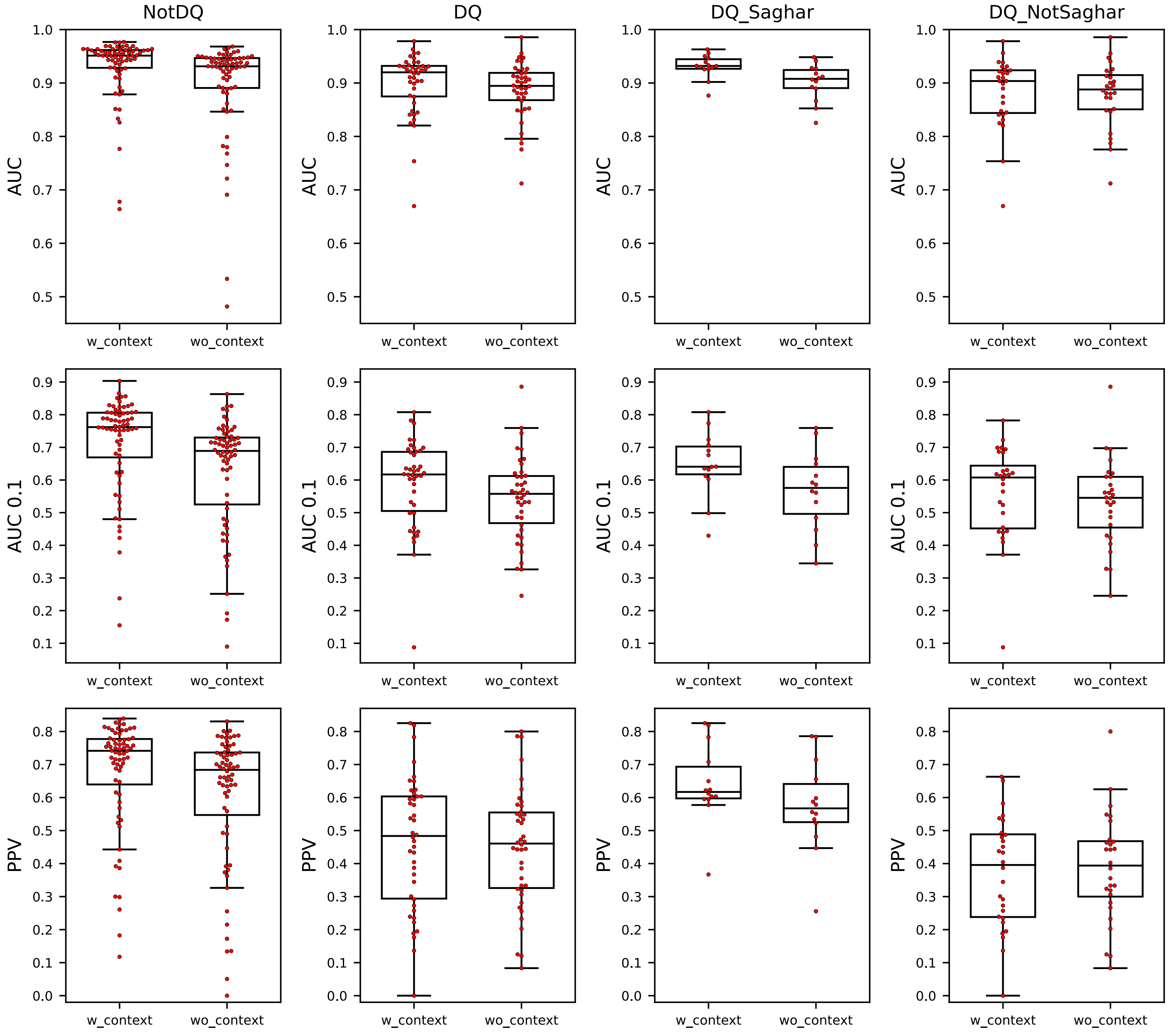

Supplementary figure 5: **Performance comparison of models trained with the novel DQ data, with (w_context) and without (wo_context) context encoding**. Each point is the performance metric for a unique HLA class II molecule. The different subgroups of HLA molecules are non-DQ molecules (NotDQ, n=68), all DQ molecules (DQ, n=42), DQ molecules present in the novel data (DQ_Saghar, n=14), and DQ molecules not present in the novel data (DQ_NotSaghar, n=28). Each boxplot shows the median inside the IQR between the upper and lower quartiles, with whiskers extending to at most 1.5 times the IQR.

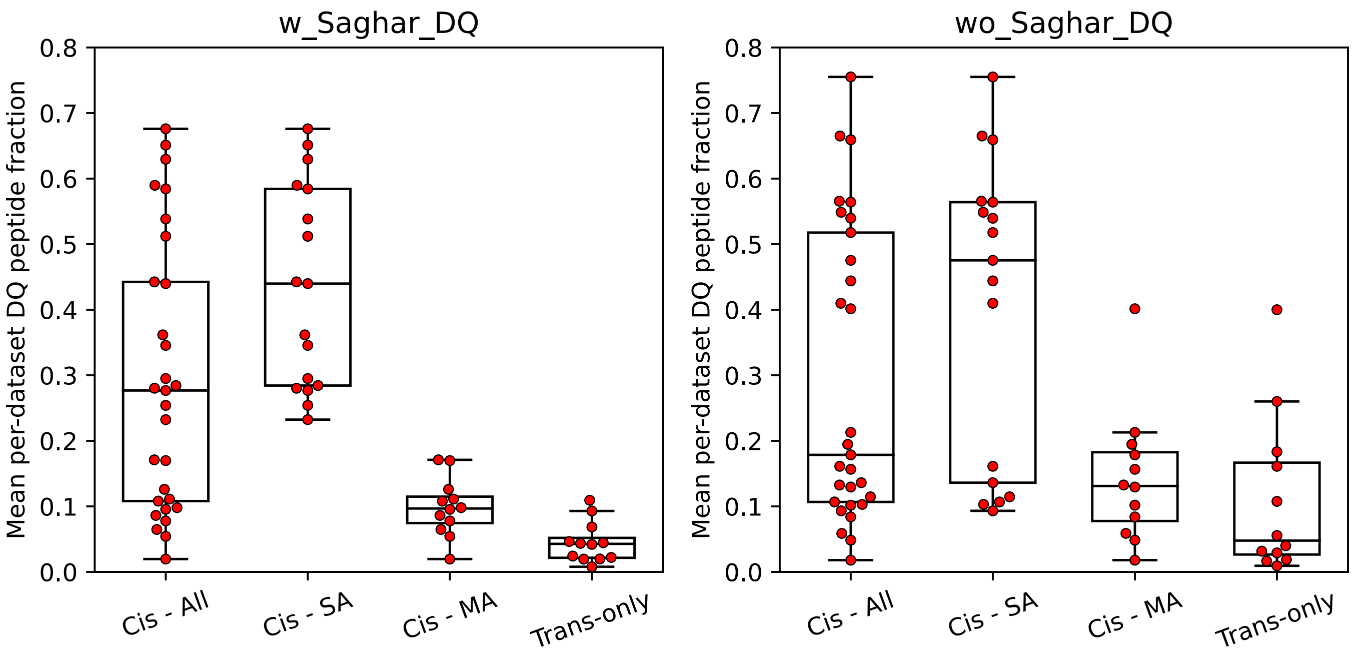

Supplementary figure 6: Peptide-count contribution of cis and trans-only molecules in the methods with (w_Saghar_DQ) and without (wo_Saghar_DQ) the novel data, taking into account pseudo-sequence overlap. Each point shows the mean per-dataset peptide fraction for a given DQ molecule. Each boxplot shows the median inside the IQR between the upper and lower quartiles, with whiskers extending to at most 1.5 times the IQR. For each method, trans-only molecules are shown in one boxplot (n=12), while cis molecules are shown in three categories, namely all cis molecules (Cis – All, n=29), cis molecules found in the DQ-SA training data or with the same pseudo-sequence as a DQ-SA molecule (Cis – SA, n=17), and cis molecules only found in the DQ-MA training data and with no pseudo-sequence overlap to cis-SA molecules (Cis – MA, n=12). Here, a significant difference was found between cis-MA and trans-only in the model with the novel data (t=3.44, p=0.002, n=12 cis-MA molecules and n=12 trans-only molecules, two-sided t-test).

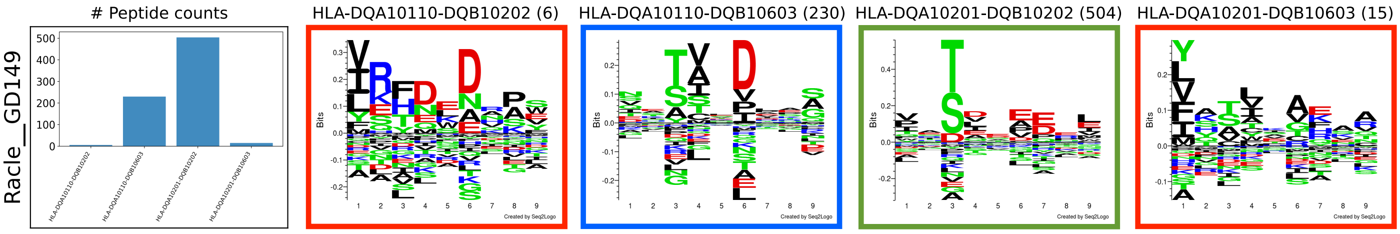

Supplementary figure 7: DQ motif deconvolution of the Racle__GD149 dataset. The logo frame colors indicate three types of DQ molecules, namely cis-molecules present in the DQ-SA training data (green), cis molecules present in the DQ-MA training data (blue), and trans-only molecules (red).

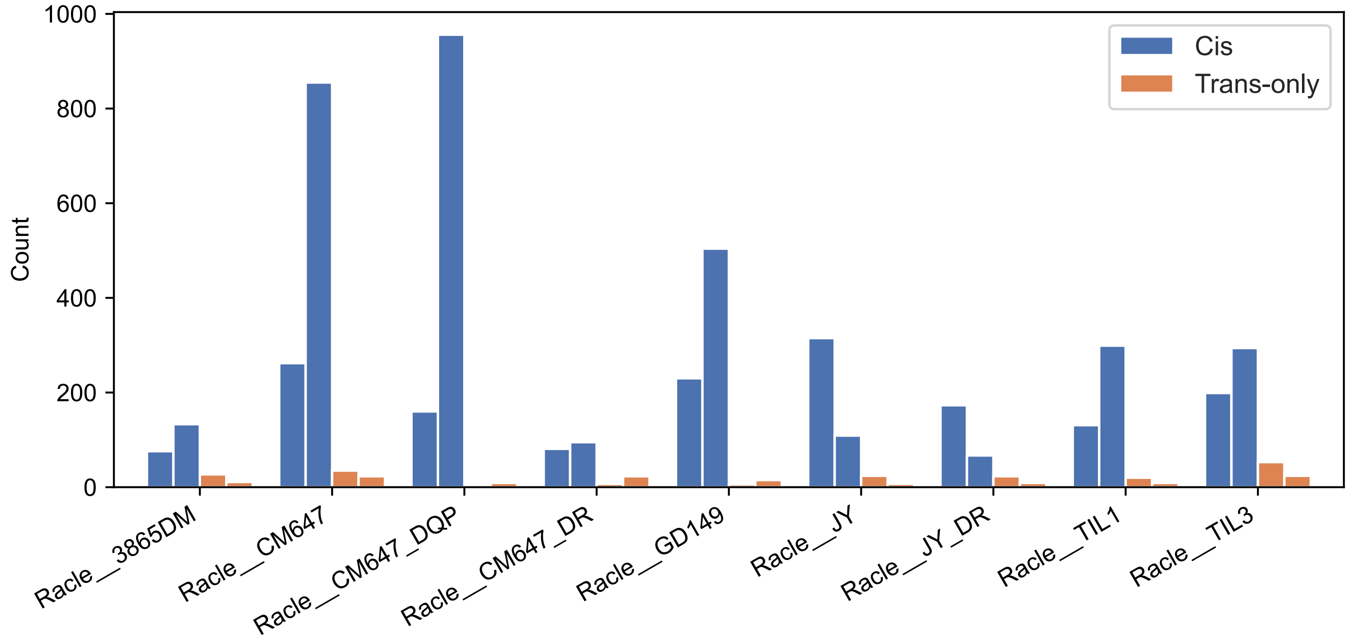

Supplementary figure 8: DQ ligand count distributions in DQ-heterozygous cell lines with both cis and trans-only molecules. Only cell lines with at least 100 DQ-annotated peptides excluding trash are included. The counts are shown for the individual molecules, colored by category (cis: blue and trans-only: orange). If we assume that the relative expression from each chromosome is linked to the observed peptide counts for the cis variants, then the trans-only variants (if they were functional) should in principle have peptide counts corresponding to at least the cis variant with the lowest count. However, this is not what we observe, as the cis variant with the lowest count always has a much larger peptide count than both trans-only variants.

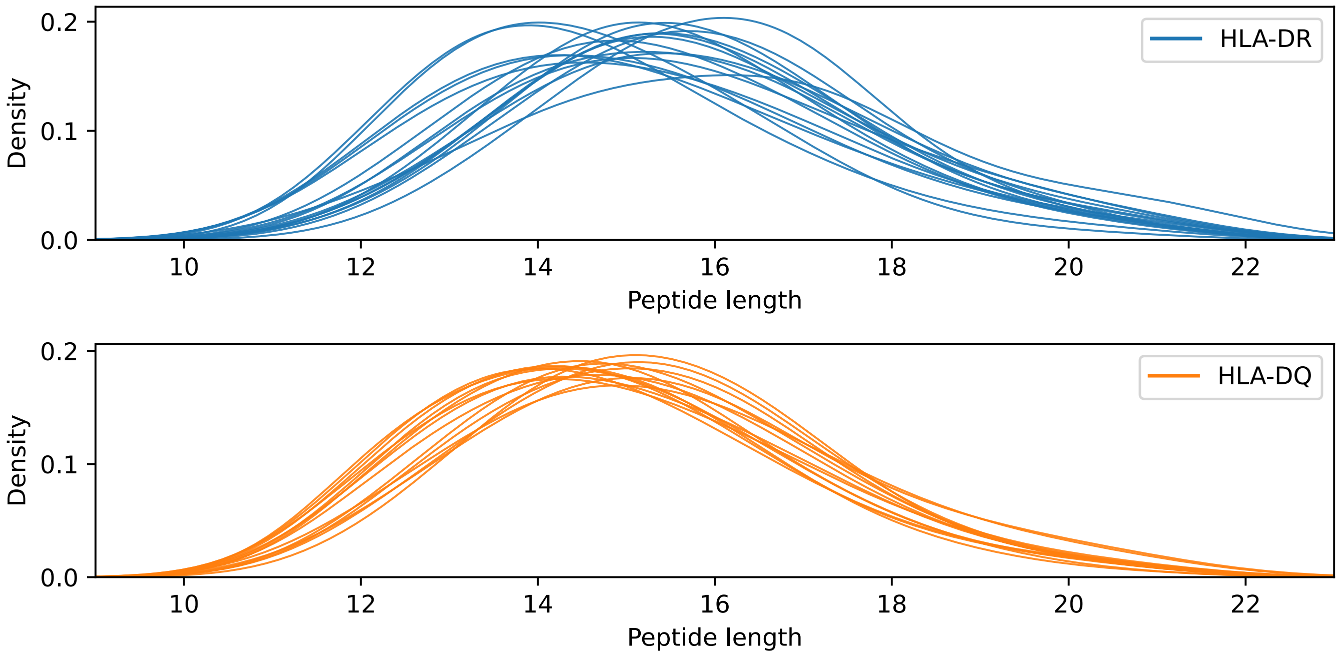

Supplementary figure 9: Kernel density plots of length distributions of peptides extracted from the cell lines used in the novel data. Each curve corresponds to the peptide length distribution for an HLA-DR (top) or HLA-DQ (bottom) molecule. The DR distributions are based on the immunopeptidomics data from supplementary table 4 in Kaabinejadian et al. 2022, which were run through the motif deconvolution method developed in this manuscript.

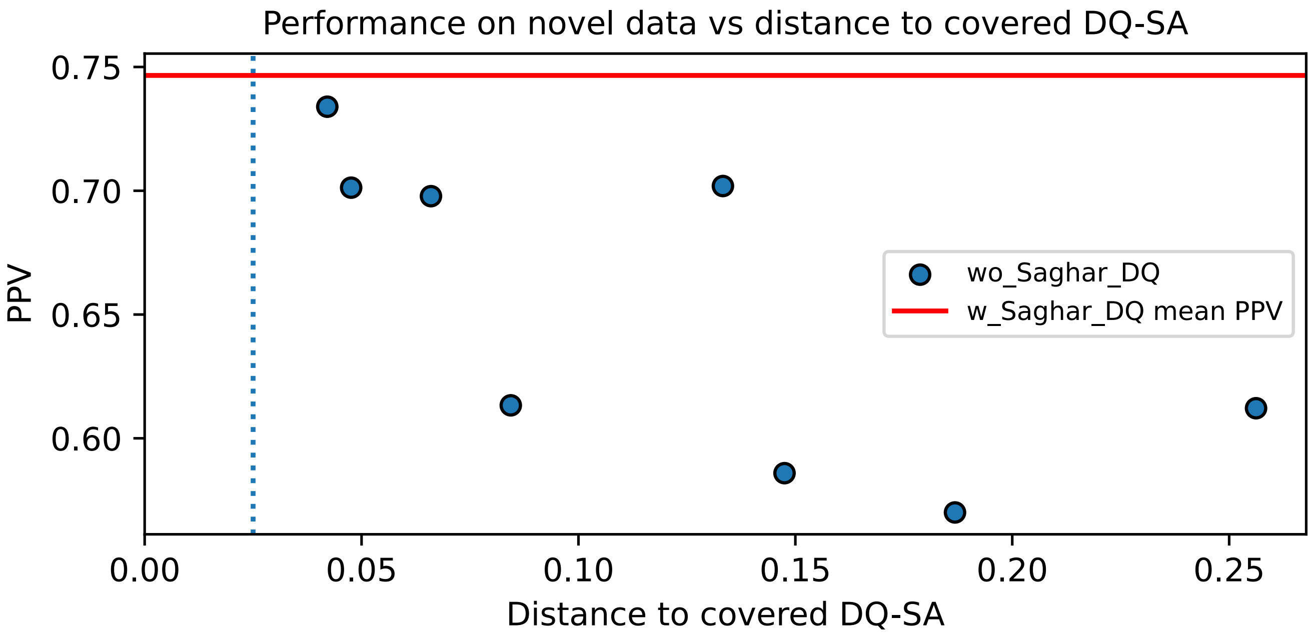

Supplementary figure 10: Performance vs distance analysis for the model trained without the novel data (wo_Saghar_DQ). Each point shows the PPV performance evaluated on the novel data for a DQ molecule not present in the DQ-SA training data of the wo_Saghar_DQ method, as a function of the distance to the training data DQ-SA molecules. The red line shows the mean performance of the model trained with the novel data (w_Saghar_DQ) on the same set of DQ molecules, evaluated on the novel data.

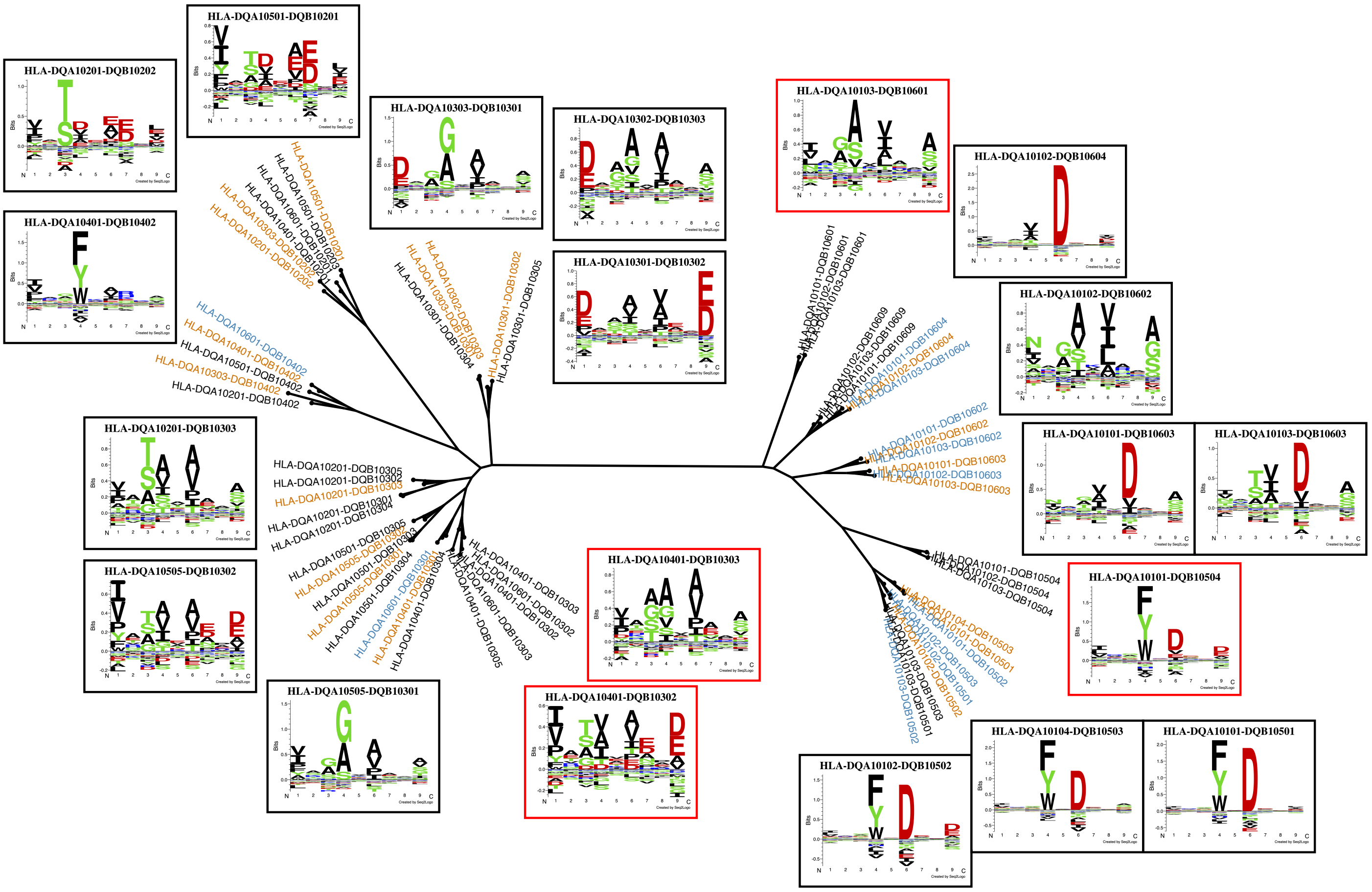

Supplementary figure 11: Sequence-based clustering of DQ molecules. The tree is based on 61 DQ molecules including the 14 molecules described by the novel data. Orange molecules are covered by the method including the novel data with at least 100 peptides, and blue molecules are within a distance 0.025 of an orange molecule. Black molecules are non-covered (i.e. have peptide count less than 100 and have distance greater than 0.025 to an orange molecule). Logos in black frames correspond to orange molecules. Logos in red frames correspond to molecules from branches with clusters of non-covered (black) molecules. The phylogenetic tree was constructed from the DQ pseudo-sequences using ClustalW. Logos were constructed from the top 1% of 100,000 random 13-17 mer peptides.

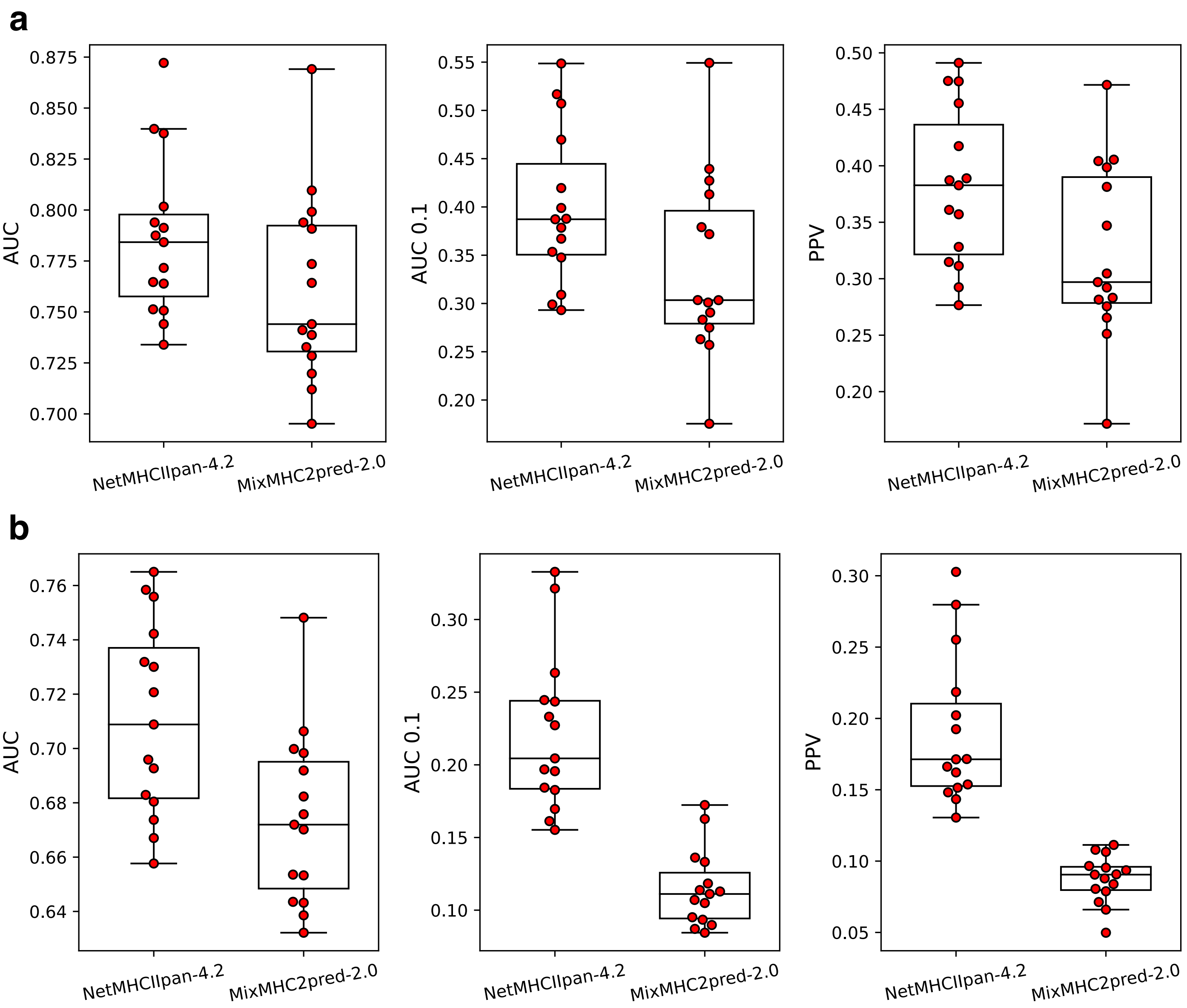

Supplementary figure 12: Benchmark against MixMHC2pred-2.0 in terms of AUC, AUC 0.1 and PPV, with both methods using peptide context encoding. Each point is the performance metric for a given sample. Each boxplot (n=15 samples in all cases) shows the median inside the IQR between the upper and lower quartiles, with whiskers extending to at most 1.5 times the IQR. **a** Performance per sample calculated on the entire data. **b** Performance per sample calculated on the union of DQ-annotated peptides between the two methods.

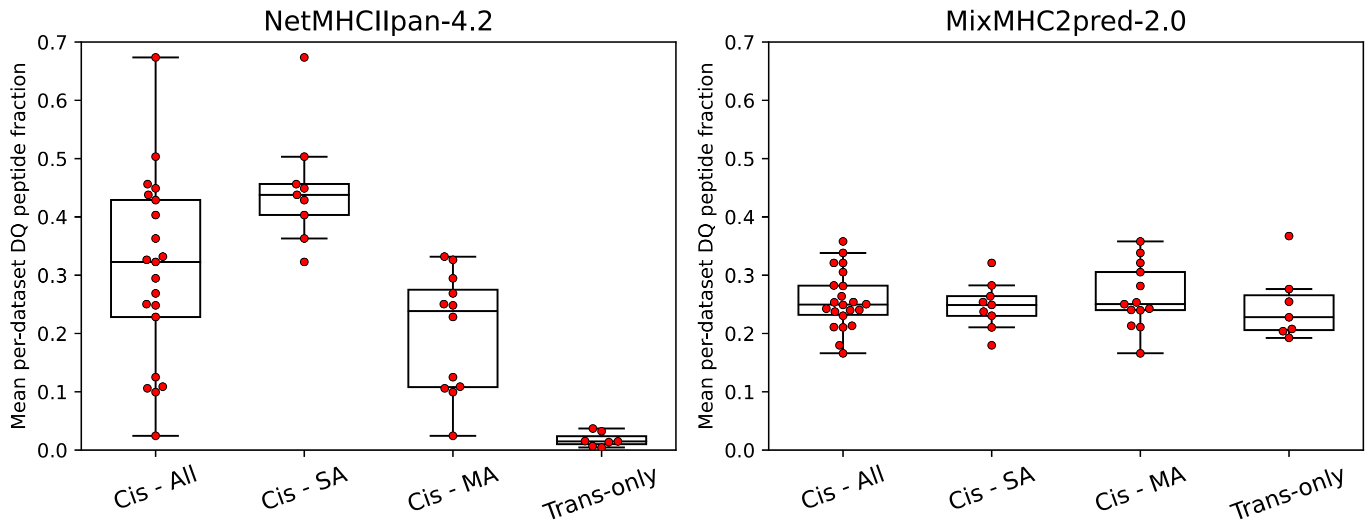

Supplementary figure 13A: Peptide-count contribution of cis and trans-only molecules predicted by NetMHCIIpan-4.2 and MixMHC2pred-2.0 on DQ-heterozygous data from Marcu et al. 2021. Each point shows the mean per-dataset peptide fraction for a given DQ molecule. Each boxplot shows the median inside the IQR between the upper and lower quartiles, with whiskers extending to at most 1.5 times the IQR. For each method, trans-only molecules are shown in one boxplot (n=7), while cis molecules are shown in three categories, namely all cis molecules (Cis – All, n=21), cis molecules found in the DQ-SA training data (Cis – SA, n=9), and cis molecules only found in the DQ-MA training data (Cis – MA, n=12 for NetMHCIIpan-4.2 and n=13 for MixMHC2pred-2.0).

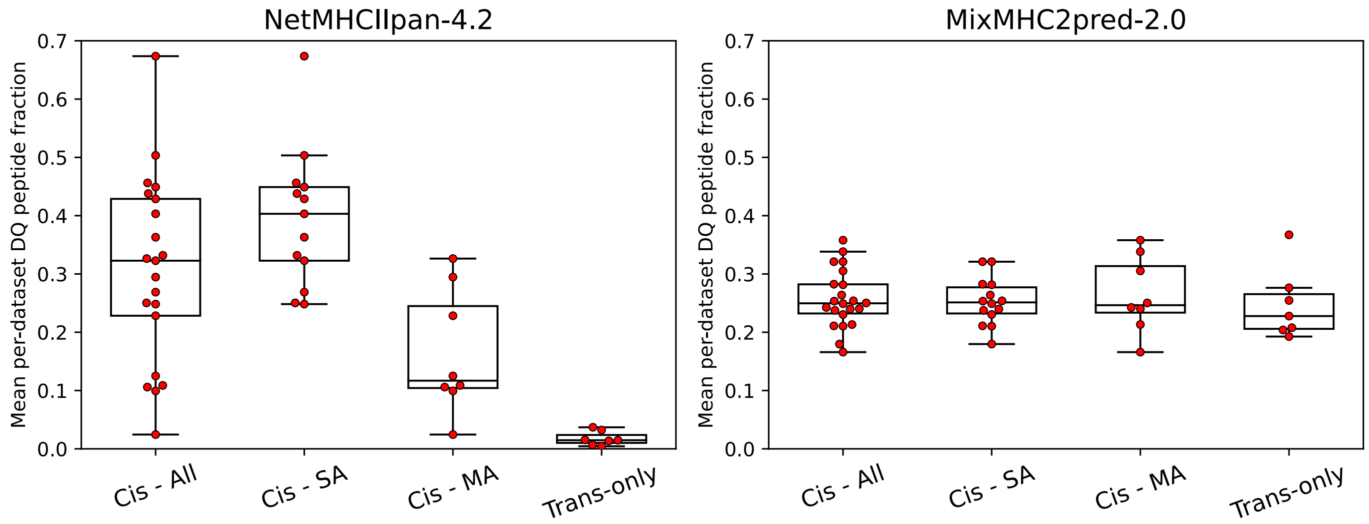

Supplementary figure 13B: Peptide-count contribution of cis and trans-only molecules predicted by NetMHCIIpan-4.2 and MixMHC2pred-2.0 on DQ-heterozygous data from Marcu et al. 2021, taking into account pseudo-sequence overlap. Each point shows the mean per-dataset peptide fraction for a given DQ molecule. Each boxplot shows the median inside the IQR between the upper and lower quartiles, with whiskers extending to at most 1.5 times the IQR. For each method, trans-only molecules are shown in one boxplot (n=7), while cis molecules are shown in three categories, namely all cis molecules (Cis – All, n=21), cis molecules found in the DQ-SA training data or with the same pseudo-sequence as a DQ-SA molecule (Cis – SA, n=13 for NetMHCIIpan-4.2 and n=14 for MixMHC2pred-2.0), and cis molecules only found in the DQ-MA training data and with no pseudo-sequence overlap to cis-SA molecules (Cis – MA, n=8). Here, a significant difference was found between cis-MA and trans-only in NetMHCIIpan-4.2 (t=3.6, p=0.003, n=8 cis-MA molecules and n=7 trans-only molecules, two-sided t-test).

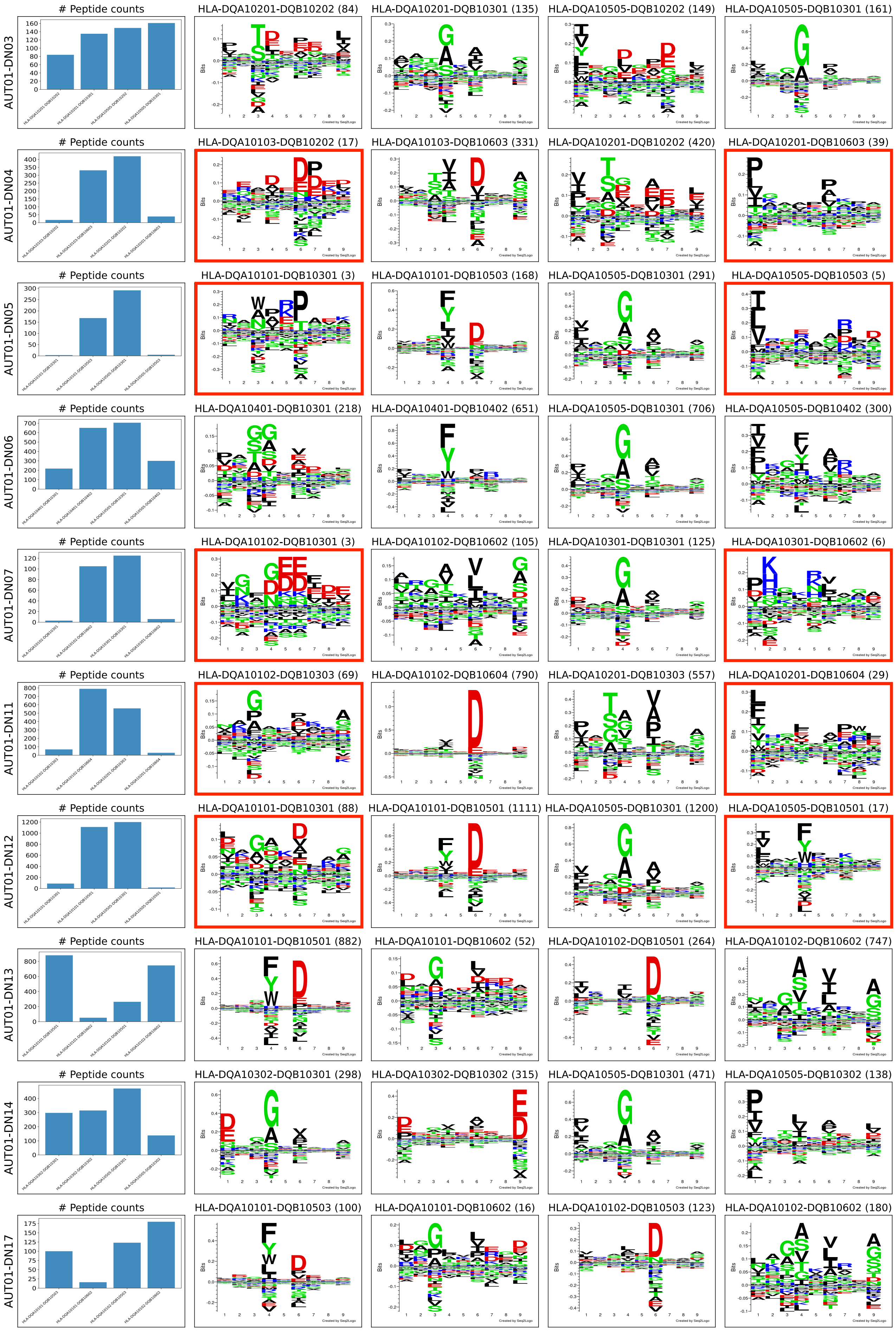

Supplementary figure 13C: DQ motif deconvolution by our method for DQ-heterozygous datasets in the benchmark data from Marcu et al. 2021. Predictions were made without peptide context encoding. Each row corresponds to a donor sample. Only peptides with percentile rank less than 10 were included in the logo plots. The number of peptides used to create each motif is shown in parenthesis above the given logo. Trans-only molecules are highlighted in red frames.

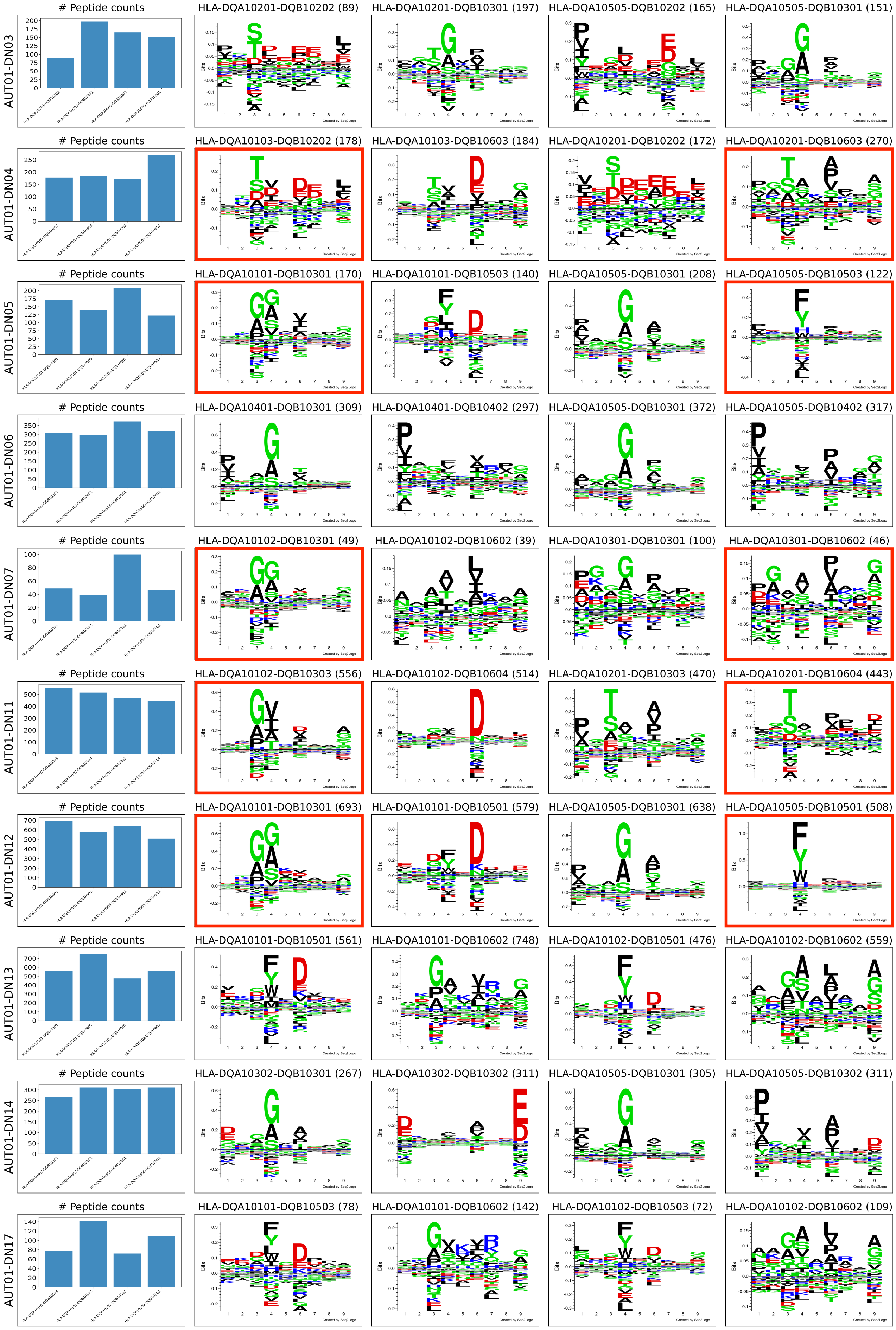

Supplementary figure 13D: DQ motif deconvolution by MixMHC2pred-2.0 for DQ-heterozygous datasets in the benchmark data from Marcu et al. 2021. Predictions were made without peptide context encoding. Each row corresponds to a donor sample. Only peptides with percentile rank less than 10 were included in the logo plots. The number of peptides used to create each motif is shown in parenthesis above the given logo. Trans-only molecules are highlighted in red frames

Supplementary tables

Supplementary table 1: Migration of peptides annotated towards HLA-DQ by the models trained with (w_Saghar) and without (wo_Saghar) the novel data.

**Peptides uniquely assigned to HLA-DQ in w_Saghar method**	**Distribution of peptide annotations in wo_Saghar method**		
	**HLA-DP**	**HLA-DR**	**Trash**
9309	1816	4688	2805
**Peptides uniquely assigned to HLA-DQ in wo_Saghar method**	**Distribution of peptide annotations in w_Saghar method**		
	**HLA-DP**	**HLA-DR**	**Trash**
4316	609	2629	1078

Supplementary table 2: Overview of peptides assigned to DQ molecules in the methods with (w_Saghar) and without (wo_Saghar) the novel data. Trash peptides with percentile rank greater than 20 are not included in the metrics. A bold value indicates either a higher average peptide count or a lower mean/median percentile rank in a given method.

	Average number of peptides per dataset		Average of per-dataset mean percentile rank		Median of per-dataset median percentile rank	
**Molecule**	**w_Saghar**	**wo_Saghar**	**w_Saghar**	**wo_Saghar**	**w_Saghar**	**wo_Saghar**
HLA-DQA1*01:01-DQB1*02:01	6.00	**7.50**	**3.2818**	3.7402	**0.6713**	0.8226
HLA-DQA1*01:01-DQB1*03:01	20.00	**170.00**	**0.3084**	1.8631	**0.0086**	0.4087
HLA-DQA1*01:01-DQB1*03:03	27.00	**58.00**	**0.4281**	0.5240	**0.0078**	0.0086
HLA-DQA1*01:01-DQB1*05:01	**209.53**	155.06	2.6547	**1.1562**	1.1783	**0.1096**
HLA-DQA1*01:01-DQB1*06:02	**30.50**	27.67	1.1216	**0.9356**	**0.2011**	0.2064
HLA-DQA1*01:01-DQB1*06:03	26.40	**59.20**	**1.2573**	2.6273	**0.1503**	2.1822
HLA-DQA1*01:02-DQB1*02:02	13.00	**28.75**	**0.1723**	1.3248	**0.0073**	0.0094
HLA-DQA1*01:02-DQB1*03:01	53.00	**99.00**	**0.3111**	1.5251	**0.0083**	0.0552
HLA-DQA1*01:02-DQB1*03:02	6.50	**8.50**	**0.3849**	0.8472	**0.0073**	0.0078
HLA-DQA1*01:02-DQB1*05:01	27.33	**43.33**	2.2276	**0.8075**	0.1011	**0.0092**
HLA-DQA1*01:02-DQB1*05:02	**512.20**	368.00	2.7410	**1.1782**	0.6769	**0.0513**
HLA-DQA1*01:02-DQB1*06:02	550.88	**551.88**	4.1209	**3.8584**	1.9511	**1.5487**
HLA-DQA1*01:02-DQB1*06:04	**1548.50**	1520.00	**1.8068**	1.9438	**0.3963**	0.4895
HLA-DQA1*01:03-DQB1*03:02	23.50	**54.50**	**0.3737**	0.9327	**0.0071**	0.0091
HLA-DQA1*01:03-DQB1*05:01	6.60	**12.80**	0.6552	**0.3573**	**0.0140**	0.0165
HLA-DQA1*01:03-DQB1*05:03	**2.00**	1.50	4.2220	**0.0102**	4.2220	**0.0102**
HLA-DQA1*01:03-DQB1*06:03	**353.10**	325.60	**2.6588**	3.0594	**0.5745**	0.5782
HLA-DQA1*01:04-DQB1*05:03	**549.00**	406.33	2.8193	**0.7154**	1.7952	**0.2739**
HLA-DQA1*01:04-DQB1*06:03	3.00	**3.50**	**3.2792**	3.5277	4.1573	**2.3288**
HLA-DQA1*01:05-DQB1*05:01	**89.00**	27.00	2.5292	**1.4787**	1.2150	**0.0631**
HLA-DQA1*01:05-DQB1*06:03	27.00	**51.00**	**0.6273**	0.9981	**0.0100**	0.0225
HLA-DQA1*01:10-DQB1*02:02	6.00	**32.00**	**0.0063**	0.0663	**0.0067**	0.0073
HLA-DQA1*01:10-DQB1*05:01	17.00	**22.00**	**0.0983**	0.9877	**0.0077**	0.0085
HLA-DQA1*01:10-DQB1*06:03	**205.00**	189.00	**1.9616**	1.9742	0.5409	**0.4139**
HLA-DQA1*02:01-DQB1*02:01	**75.67**	73.33	**2.2314**	2.3259	0.5515	**0.3520**
HLA-DQA1*02:01-DQB1*02:02	1188.91	**1207.82**	3.4357	**2.9958**	**1.1275**	1.1813
HLA-DQA1*02:01-DQB1*03:01	8.00	**10.50**	6.1621	**4.9487**	**2.2676**	2.6416
HLA-DQA1*02:01-DQB1*03:03	**868.00**	711.00	3.8598	**2.8138**	2.0423	**0.9332**
HLA-DQA1*02:01-DQB1*05:01	11.00	**24.00**	**0.0094**	1.5804	**0.0060**	0.0080
HLA-DQA1*02:01-DQB1*05:02	**18.33**	15.67	**0.0088**	2.0617	**0.0059**	0.0083

HLA-DQA1*02:01-DQB1*06:02	6.00	**7.00**	1.0585	**0.0980**	0.0355	**0.0091**
HLA-DQA1*02:01-DQB1*06:03	15.00	15.00	**0.0088**	0.2958	0.0085	**0.0072**
HLA-DQA1*03:01-DQB1*02:01	**14.67**	14.33	3.0440	**2.0955**	**0.0953**	0.9899
HLA-DQA1*03:01-DQB1*03:01	**54.67**	16.83	3.5246	**0.7732**	1.1628	**0.0092**
HLA-DQA1*03:01-DQB1*03:02	**152.86**	92.64	4.2735	**1.5354**	2.0231	**0.1016**
HLA-DQA1*03:01-DQB1*06:02	**4.00**	1.00	2.3313	**0.4498**	**0.2132**	0.4498
HLA-DQA1*03:01-DQB1*06:03	8.00	**10.00**	0.6198	**0.0115**	0.0270	**0.0088**
HLA-DQA1*03:02-DQB1*03:03	**1565.00**	1003.00	3.3282	**2.6386**	1.4368	**0.6301**
HLA-DQA1*03:03-DQB1*02:02	**48.33**	15.00	0.8371	**0.1817**	**0.0096**	0.0109
HLA-DQA1*03:03-DQB1*03:01	**593.25**	389.50	3.0677	**1.0865**	0.8015	**0.0512**
HLA-DQA1*03:03-DQB1*04:02	**82.00**	17.00	**0.8113**	1.2414	**0.0109**	0.0204
HLA-DQA1*03:03-DQB1*05:01	**9.00**	4.00	**0.0143**	0.0253	**0.0085**	0.0258
HLA-DQA1*04:01-DQB1*03:01	96.67	**371.00**	**1.4585**	3.5739	**0.0179**	1.5131
HLA-DQA1*04:01-DQB1*04:02	**967.60**	801.80	3.0511	**2.3695**	1.4733	**0.5541**
HLA-DQA1*05:01-DQB1*02:01	344.00	**347.79**	**2.2603**	2.6852	**0.2518**	0.3707
HLA-DQA1*05:01-DQB1*03:01	1008.50	1008.50	4.6531	**4.3624**	1.7700	**1.6834**
HLA-DQA1*05:01-DQB1*03:02	19.67	**22.00**	3.5080	**2.4423**	**0.2787**	0.5770
HLA-DQA1*05:01-DQB1*05:01	2.00	**4.00**	**0.0071**	0.0238	**0.0070**	0.0082
HLA-DQA1*05:05-DQB1*02:02	**64.00**	62.40	2.4021	**2.1639**	**0.1911**	0.3171
HLA-DQA1*05:05-DQB1*03:01	577.55	**594.90**	**3.3196**	3.8926	**1.4065**	1.8265
HLA-DQA1*05:05-DQB1*03:02	18.67	**25.33**	**1.2711**	1.5989	0.0884	**0.0495**
HLA-DQA1*05:05-DQB1*05:02	**24.00**	9.00	0.0093	**0.0068**	**0.0064**	0.0071

Supplementary table 3: Overview of consistency analysis in the methods with (w_Saghar) and without (wo_Saghar) the novel data. The molecules are sorted in descending order by the difference in mean consistency. Further, the metrics are calculated on the peptide sets used in the consistency analysis, with the union of identified trash peptides removed. As such, the metrics regarding differences in percentile ranks may not correspond one-to-one with the values in supplementary table 4.

Molecules	Difference in mean consistency (w_Saghar - wo_Saghar)	Number of cell lines with at least 20 peptides in both methods	Number of cell lines with larger peptide count in w_Saghar	Number of cell lines with lower mean %rank in w_Saghar	Fraction of cell lines with either larger peptide count or lower mean %rank in w_Saghar
**HLA-DQA1*03:01-DQB1*03:02**	**0.148**	**6**	**6**	**1**	**1.000**
**HLA-DQA1*03:01-DQB1*03:01**	**0.147**	**2**	**2**	**0**	**1.000**
**HLA-DQA1*03:03-DQB1*03:01**	**0.119**	**5**	**5**	**1**	**1.000**
**HLA-DQA1*01:01-DQB1*05:01**	**0.079**	**10**	**10**	**3**	**1.000**
**HLA-DQA1*04:01-DQB1*04:02**	**0.039**	**5**	**3**	**2**	**0.600**
**HLA-DQA1*01:02-DQB1*05:02**	**0.037**	**5**	**5**	**1**	**1.000**
**HLA-DQA1*02:01-DQB1*03:03**	**0.035**	**2**	**2**	**1**	**1.000**
**HLA-DQA1*01:10-DQB1*06:03**	**0.034**	**2**	**2**	**1**	**1.000**
**HLA-DQA1*05:05-DQB1*03:01**	**0.027**	**16**	**2**	**8**	**0.625**
**HLA-DQA1*01:02-DQB1*06:02**	**0.020**	**7**	**3**	**1**	**0.429**
**HLA-DQA1*02:01-DQB1*02:01**	**0.017**	**3**	**2**	**1**	**0.667**
**HLA-DQA1*05:01-DQB1*02:01**	**0.016**	**12**	**4**	**9**	**0.917**
**HLA-DQA1*05:05-DQB1*02:02**	**0.014**	**3**	**0**	**2**	**0.667**
**HLA-DQA1*02:01-DQB1*02:02**	**0.010**	**9**	**1**	**8**	**1.000**
**HLA-DQA1*05:05-DQB1*03:02**	**0.005**	**2**	**1**	**1**	**1.000**
**HLA-DQA1*05:01-DQB1*03:01**	**0.002**	**4**	**3**	**0**	**0.750**
HLA-DQA1*01:02-DQB1*06:04	-0.002	2	1	2	1.000
HLA-DQA1*01:03-DQB1*03:02	-0.008	2	0	2	1.000
HLA-DQA1*01:03-DQB1*06:03	-0.009	7	5	4	0.857
HLA-DQA1*01:01-DQB1*06:03	-0.027	2	0	2	1.000
HLA-DQA1*04:01-DQB1*03:01	-0.031	3	0	3	1.000

Supplementary table 4: Overview of DQ-heterozygous datasets used in the cis vs trans-only DQ analysis, along with their DQ HLA typing. Molecules marked in red are trans-only heterodimers. Molecules in bold are part of the DQ-SA training data.

Dataset	DQ heterodimers			
**Racle__3808_HMC**	DQA1*02:01-DQB1*02:01	**DQA1*02:01-DQB1*02:02**	**DQA1*05:01-DQB1*02:01**	DQA1*05:01-DQB1*02:02
**Racle__3830NJF**	DQA1*03:01-DQB1*03:01	**DQA1*03:01-DQB1*03:02**	**DQA1*05:05-DQB1*03:01**	DQA1*05:05-DQB1*03:02
**Racle__3830_NJF_DQP**	DQA1*03:01-DQB1*03:01	**DQA1*03:01-DQB1*03:02**	**DQA1*05:05-DQB1*03:01**	DQA1*05:05-DQB1*03:02
**Racle__3865DM**	DQA1*01:01-DQB1*03:03	**DQA1*01:01-DQB1*05:01**	**DQA1*02:01-DQB1*03:03**	DQA1*02:01-DQB1*05:01
**Racle__3869_GA**	DQA1*03:01-DQB1*03:01	**DQA1*03:01-DQB1*03:02**	**DQA1*05:05-DQB1*03:01**	DQA1*05:05-DQB1*03:02
**Racle__3869_GA_DQP**	DQA1*03:01-DQB1*03:01	**DQA1*03:01-DQB1*03:02**	**DQA1*05:05-DQB1*03:01**	DQA1*05:05-DQB1*03:02
**Racle__3912BAM**	DQA1*03:01-DQB1*02:01	**DQA1*03:01-DQB1*03:02**	**DQA1*05:01-DQB1*02:01**	DQA1*05:01-DQB1*03:02
**Racle__3912_BAM_DR**	DQA1*03:01-DQB1*02:01	**DQA1*03:01-DQB1*03:02**	**DQA1*05:01-DQB1*02:01**	DQA1*05:01-DQB1*03:02
**Racle__3947_GA**	**DQA1*01:01-DQB1*05:01**	DQA1*01:01-DQB1*06:03	DQA1*01:03-DQB1*05:01	**DQA1*01:03-DQB1*06:03**
**Racle__4021**	DQA1*03:03-DQB1*02:02	**DQA1*03:03-DQB1*03:01**	DQA1*05:05-DQB1*02:02	**DQA1*05:05-DQB1*03:01**
**Racle__4021_DQP**	DQA1*03:03-DQB1*02:02	**DQA1*03:03-DQB1*03:01**	DQA1*05:05-DQB1*02:02	**DQA1*05:05-DQB1*03:01**
**Racle__4052_BA**	**DQA1*05:01-DQB1*02:01**	DQA1*05:01-DQB1*03:01	DQA1*05:05-DQB1*02:01	**DQA1*05:05-DQB1*03:01**
**Racle__4052_BA_DQP**	**DQA1*05:01-DQB1*02:01**	DQA1*05:01-DQB1*03:01	DQA1*05:05-DQB1*02:01	**DQA1*05:05-DQB1*03:01**
**Racle__BP455**	DQA1*01:05-DQB1*05:01	DQA1*01:05-DQB1*06:03	DQA1*01:10-DQB1*05:01	DQA1*01:10-DQB1*06:03
**Racle__CM647**	DQA1*01:02-DQB1*02:02	**DQA1*01:02-DQB1*05:02**	**DQA1*02:01-DQB1*02:02**	DQA1*02:01-DQB1*05:02
**Racle__CM647_DQP**	DQA1*01:02-DQB1*02:02	**DQA1*01:02-DQB1*05:02**	**DQA1*02:01-DQB1*02:02**	DQA1*02:01-DQB1*05:02
**Racle__CM647_DR**	DQA1*01:02-DQB1*02:02	**DQA1*01:02-DQB1*05:02**	**DQA1*02:01-DQB1*02:02**	DQA1*02:01-DQB1*05:02
**Racle__GD149**	DQA1*01:10-DQB1*02:02	DQA1*01:10-DQB1*06:03	**DQA1*02:01-DQB1*02:02**	DQA1*02:01-DQB1*06:03
**Racle__JY**	DQA1*01:03-DQB1*03:02	**DQA1*01:03-DQB1*06:03**	**DQA1*03:01-DQB1*03:02**	DQA1*03:01-DQB1*06:03
**Racle__JY_DR**	DQA1*01:03-DQB1*03:02	**DQA1*01:03-DQB1*06:03**	**DQA1*03:01-DQB1*03:02**	DQA1*03:01-DQB1*06:03
**Racle__PD42**	**DQA1*01:01-DQB1*05:01**	DQA1*01:01-DQB1*06:02	DQA1*01:02-DQB1*05:01	**DQA1*01:02-DQB1*06:02**
**Racle__RA957**	**DQA1*03:03-DQB1*03:01**	DQA1*03:03-DQB1*04:02	DQA1*04:01-DQB1*03:01	**DQA1*04:01-DQB1*04:02**
**Racle__RA957_DQP**	**DQA1*03:03-DQB1*03:01**	DQA1*03:03-DQB1*04:02	DQA1*04:01-DQB1*03:01	**DQA1*04:01-DQB1*04:02**
**Racle__RA957_DR**	**DQA1*03:03-DQB1*03:01**	DQA1*03:03-DQB1*04:02	DQA1*04:01-DQB1*03:01	**DQA1*04:01-DQB1*04:02**
**Racle__TIL1**	DQA1*01:01-DQB1*03:01	**DQA1*01:01-DQB1*05:01**	**DQA1*03:03-DQB1*03:01**	DQA1*03:03-DQB1*05:01
**Racle__TIL3**	DQA1*01:02-DQB1*03:01	**DQA1*01:02-DQB1*05:02**	**DQA1*05:05-DQB1*03:01**	DQA1*05:05-DQB1*05:02
**Ritz__DOHH2_DQ**	**DQA1*01:01-DQB1*05:01**	**DQA1*01:02-DQB1*06:02**	DQA1*01:02-DQB1*05:01	DQA1*01:01-DQB1*06:02
**Ritz__Maver_1_DQ**	**DQA1*01:01-DQB1*05:01**	**DQA1*01:03-DQB1*06:03**	DQA1*01:01-DQB1*06:03	DQA1*01:03-DQB1*05:01

Supplementary table 5: DQ molecules covered by the method including the novel data. The highlighted molecules are covered by a peptide count of at least 100, while the remaining molecules have a distance of at most 0.025 to one of the highlighted molecules.

**HLA-DQA1*01:01-DQB1*05:01**	HLA-DQA1*01:01-DQB1*05:02	HLA-DQA1*03:01-DQB1*02:02	HLA-DQA1*05:01-DQB1*03:19
**HLA-DQA1*01:01-DQB1*06:03**	HLA-DQA1*01:01-DQB1*05:03	HLA-DQA1*03:01-DQB1*03:03	HLA-DQA1*05:01-DQB1*03:22
**HLA-DQA1*01:02-DQB1*05:02**	HLA-DQA1*01:01-DQB1*06:02	HLA-DQA1*03:01-DQB1*03:19	HLA-DQA1*05:01-DQB1*03:62
**HLA-DQA1*01:02-DQB1*06:02**	HLA-DQA1*01:01-DQB1*06:04	HLA-DQA1*03:01-DQB1*03:22	HLA-DQA1*05:02-DQB1*02:01
**HLA-DQA1*01:02-DQB1*06:04**	HLA-DQA1*01:02-DQB1*05:01	HLA-DQA1*03:01-DQB1*03:62	HLA-DQA1*05:02-DQB1*02:02
**HLA-DQA1*01:03-DQB1*06:03**	HLA-DQA1*01:02-DQB1*05:03	HLA-DQA1*03:01-DQB1*03:87	HLA-DQA1*05:02-DQB1*03:01
**HLA-DQA1*01:04-DQB1*05:03**	HLA-DQA1*01:02-DQB1*05:46	HLA-DQA1*03:01-DQB1*04:01	HLA-DQA1*05:02-DQB1*03:02
**HLA-DQA1*01:10-DQB1*06:03**	HLA-DQA1*01:02-DQB1*06:03	HLA-DQA1*03:01-DQB1*04:02	HLA-DQA1*05:03-DQB1*02:01
**HLA-DQA1*02:01-DQB1*02:01**	HLA-DQA1*01:03-DQB1*05:02	HLA-DQA1*03:02-DQB1*02:01	HLA-DQA1*05:03-DQB1*02:02
**HLA-DQA1*02:01-DQB1*02:02**	HLA-DQA1*01:03-DQB1*06:02	HLA-DQA1*03:02-DQB1*02:02	HLA-DQA1*05:03-DQB1*03:01
**HLA-DQA1*02:01-DQB1*03:03**	HLA-DQA1*01:03-DQB1*06:04	HLA-DQA1*03:02-DQB1*03:01	HLA-DQA1*05:03-DQB1*03:02
**HLA-DQA1*03:01-DQB1*03:01**	HLA-DQA1*01:04-DQB1*05:01	HLA-DQA1*03:02-DQB1*03:02	HLA-DQA1*05:03-DQB1*03:22
**HLA-DQA1*03:01-DQB1*03:02**	HLA-DQA1*01:04-DQB1*05:02	HLA-DQA1*03:02-DQB1*03:22	HLA-DQA1*05:05-DQB1*02:01
**HLA-DQA1*03:02-DQB1*03:03**	HLA-DQA1*01:04-DQB1*06:02	HLA-DQA1*03:02-DQB1*04:01	HLA-DQA1*05:05-DQB1*03:22
**HLA-DQA1*03:03-DQB1*02:02**	HLA-DQA1*01:04-DQB1*06:03	HLA-DQA1*03:02-DQB1*04:02	HLA-DQA1*05:06-DQB1*03:01
**HLA-DQA1*03:03-DQB1*03:01**	HLA-DQA1*01:04-DQB1*06:04	HLA-DQA1*03:03-DQB1*02:01	HLA-DQA1*05:06-DQB1*03:02
**HLA-DQA1*03:03-DQB1*04:02**	HLA-DQA1*01:05-DQB1*05:01	HLA-DQA1*03:03-DQB1*03:02	HLA-DQA1*05:08-DQB1*02:01
**HLA-DQA1*04:01-DQB1*03:01**	HLA-DQA1*01:05-DQB1*05:02	HLA-DQA1*03:03-DQB1*03:03	HLA-DQA1*05:08-DQB1*03:01
**HLA-DQA1*04:01-DQB1*04:02**	HLA-DQA1*01:05-DQB1*05:03	HLA-DQA1*03:03-DQB1*03:22	HLA-DQA1*05:08-DQB1*03:02
**HLA-DQA1*05:01-DQB1*02:01**	HLA-DQA1*01:05-DQB1*06:02	HLA-DQA1*03:03-DQB1*04:01	HLA-DQA1*06:01-DQB1*03:01
**HLA-DQA1*05:01-DQB1*03:01**	HLA-DQA1*01:05-DQB1*06:03	HLA-DQA1*04:01-DQB1*03:22	HLA-DQA1*06:01-DQB1*03:22
**HLA-DQA1*05:05-DQB1*02:02**	HLA-DQA1*01:05-DQB1*06:04	HLA-DQA1*04:01-DQB1*04:01	HLA-DQA1*06:01-DQB1*04:01
**HLA-DQA1*05:05-DQB1*03:01**	HLA-DQA1*02:01-DQB1*03:87	HLA-DQA1*05:01-DQB1*02:02	HLA-DQA1*06:01-DQB1*04:02
**HLA-DQA1*05:05-DQB1*03:02**	HLA-DQA1*03:01-DQB1*02:01	HLA-DQA1*05:01-DQB1*03:02	

Supplementary table 6: Prevalent DQ molecules with distance greater than 0.025 to molecules covered by the method including the novel data.

HLA-DQA1*01:01-DQB1*05:04	HLA-DQA1*01:04-DQB1*06:09	HLA-DQA1*04:01-DQB1*02:01	HLA-DQA1*05:03-DQB1*03:03
HLA-DQA1*01:01-DQB1*06:01	HLA-DQA1*01:05-DQB1*06:01	HLA-DQA1*04:01-DQB1*02:02	HLA-DQA1*05:03-DQB1*03:04
HLA-DQA1*01:01-DQB1*06:05	HLA-DQA1*02:01-DQB1*03:01	HLA-DQA1*04:01-DQB1*03:02	HLA-DQA1*05:03-DQB1*03:05
HLA-DQA1*01:01-DQB1*06:09	HLA-DQA1*02:01-DQB1*03:02	HLA-DQA1*04:01-DQB1*03:03	HLA-DQA1*05:03-DQB1*04:01
HLA-DQA1*01:02-DQB1*05:04	HLA-DQA1*02:01-DQB1*03:04	HLA-DQA1*04:01-DQB1*03:04	HLA-DQA1*05:03-DQB1*04:02
HLA-DQA1*01:02-DQB1*06:01	HLA-DQA1*02:01-DQB1*03:05	HLA-DQA1*04:01-DQB1*03:05	HLA-DQA1*05:05-DQB1*03:03
HLA-DQA1*01:02-DQB1*06:05	HLA-DQA1*02:01-DQB1*03:19	HLA-DQA1*05:01-DQB1*02:03	HLA-DQA1*05:05-DQB1*03:04
HLA-DQA1*01:02-DQB1*06:09	HLA-DQA1*02:01-DQB1*03:22	HLA-DQA1*05:01-DQB1*03:03	HLA-DQA1*05:05-DQB1*03:05
HLA-DQA1*01:03-DQB1*05:01	HLA-DQA1*02:01-DQB1*03:62	HLA-DQA1*05:01-DQB1*03:04	HLA-DQA1*05:05-DQB1*04:01
HLA-DQA1*01:03-DQB1*05:03	HLA-DQA1*02:01-DQB1*04:01	HLA-DQA1*05:01-DQB1*03:05	HLA-DQA1*05:05-DQB1*04:02
HLA-DQA1*01:03-DQB1*05:04	HLA-DQA1*02:01-DQB1*04:02	HLA-DQA1*05:01-DQB1*03:87	HLA-DQA1*05:08-DQB1*03:03
HLA-DQA1*01:03-DQB1*06:01	HLA-DQA1*03:01-DQB1*03:04	HLA-DQA1*05:01-DQB1*04:01	HLA-DQA1*06:01-DQB1*02:01
HLA-DQA1*01:03-DQB1*06:05	HLA-DQA1*03:01-DQB1*03:05	HLA-DQA1*05:01-DQB1*04:02	HLA-DQA1*06:01-DQB1*02:02
HLA-DQA1*01:03-DQB1*06:09	HLA-DQA1*03:02-DQB1*03:04	HLA-DQA1*05:02-DQB1*03:03	HLA-DQA1*06:01-DQB1*03:02
HLA-DQA1*01:04-DQB1*06:01	HLA-DQA1*03:02-DQB1*03:05	HLA-DQA1*05:02-DQB1*04:02	HLA-DQA1*06:01-DQB1*03:03

Supplementary table 7: Overview of donor cell line and their HLA typing in the benchmark data taken from Marcu et al. 2021.

Sample ID	HLA typing
AUT01-DN02	DRB1*04:01,DRB4*01:03,DQA1*03:01-DQB1*03:01,DQA1*03:02-DQB1*03:01,DPA1*01:03-DPB1*04:01,DPA1*01:03-DPB1*04:02
AUT01-DN03	DRB1*07:01,DRB1*11:03,DRB3*02:02,DRB4*01:01,DQA1*02:01-DQB1*02:02,DQA1*02:01-DQB1*03:01,DQA1*05:05-DQB1*02:02,DQA1*05:05-DQB1*03:01,DPA1*01:03-DPB1*03:01,DPA1*01:03-DPB1*04:01
AUT01-DN04	DRB1*07:01,DRB1*13:01,DRB3*01:01,DRB4*01:01,DQA1*01:03-DQB1*02:02,DQA1*01:03-DQB1*06:03,DQA1*02:01-DQB1*02:02,DQA1*02:01-DQB1*06:03,DPA1*01:03-DPB1*03:01,DPA1*01:03-DPB1*04:01,DPA1*02:02-DPB1*03:01,DPA1*02:02-DPB1*04:01
AUT01-DN05	DRB1*11:01,DRB1*14:54,DRB3*02:02,DQA1*01:01-DQB1*03:01,DQA1*01:01-DQB1*05:03,DQA1*05:05-DQB1*03:01,DQA1*05:05-DQB1*05:03,DPA1*01:03-DPB1*04:01
AUT01-DN06	DRB1*08:01,DRB1*13:03,DRB3*01:01,DQA1*04:01-DQB1*03:01,DQA1*04:01-DQB1*04:02,DQA1*05:05-DQB1*03:01,DQA1*05:05-DQB1*04:02,DPA1*01:03-DPB1*04:01,DPA1*01:03-DPB1*04:02
AUT01-DN08	DRB1*13:03,DRB1*14:01,DQA1*05:05-DQB1*03:01
AUT01-DN09	DRB1*07:01,DRB4*01:03,DQA1*02:01-DQB1*02:02,DPA1*01:03-DPB1*03:01,DPA1*01:03-DPB1*04:02
AUT01-DN11	DRB1*07:01,DRB1*13:02,DRB3*03:01,DRB4*01:03,DQA1*01:02-DQB1*03:03,DQA1*01:02-DQB1*06:04,DQA1*02:01-DQB1*03:03,DQA1*02:01-DQB1*06:04,DPA1*01:03-DPB1*03:01,DPA1*01:03-DPB1*04:01
AUT01-DN12	DRB1*01:01,DRB1*12:01,DRB3*02:02,DQA1*01:01-DQB1*03:01,DQA1*01:01-DQB1*05:01,DQA1*05:05-DQB1*03:01,DQA1*05:05-DQB1*05:01,DPA1*01:03-DPB1*02:01,DPA1*01:03-DPB1*09:01,DPA1*02:01-DPB1*02:01,DPA1*02:01-DPB1*09:01
AUT01-DN13	DRB1*10:01,DRB1*15:01,DRB5*01:01,DQA1*01:01-DQB1*05:01,DQA1*01:01-DQB1*06:02,DQA1*01:02-DQB1*05:01,DQA1*01:02-DQB1*06:02,DPA1*01:03-DPB1*02:01,DPA1*01:03-DPB1*04:02
AUT01-DN14	DRB1*04:01,DRB1*13:03,DRB3*01:01,DRB4*01:03,DQA1*03:02-DQB1*03:01,DQA1*03:02-DQB1*03:02,DQA1*05:05-DQB1*03:01,DQA1*05:05-DQB1*03:02,DPA1*01:03-DPB1*02:01,DPA1*02:01-DPB1*02:01
AUT01-DN15	DRB1*03:01,DRB1*11:01,DRB3*01:01,DRB3*02:02,DQA1*05:01-DQB1*02:01,DQA1*05:01-DQB1*03:01,DPA1*01:03-DPB1*01:01,DPA1*01:03-DPB1*03:01,DPA1*02:01-DPB1*01:01,DPA1*02:01-DPB1*03:01
AUT01-DN16	DRB1*03:01,DRB1*04:05,DQA1*03:02-DQB1*02:01,DQA1*03:02-DQB1*02:02,DQA1*05:01-DQB1*02:01,DQA1*05:01-DQB1*02:02
AUT01-DN17	DRB1*14:54,DRB1*15:01,DRB3*02:02,DRB5*01:01,DQA1*01:01-DQB1*05:03,DQA1*01:01-DQB1*06:02,DQA1*01:02-DQB1*05:03,DQA1*01:02-DQB1*06:02,DPA1*01:03-DPB1*02:01
