# Supplementary material for: Machine learning reveals limited contribution of trans-only encoded variants to the HLA-DQ immunopeptidome
Source: Commun Biol. 2023 Apr 21;6:442. doi: 10.1038/s42003-023-04749-7 (PMC10121683; doi:10.1038/s42003-023-04749-7)
Supplement: Supplementary file 2 — Description of Additional Supplementary Files [file 42003_2023_4749_MOESM2_ESM.docx]

**Description of Additional Supplementary Files**

**File name:** Supplementary Data 1

**Description:** Overview of HLA types per EL dataset used to train our method.

**File name:** Supplementary Data 2

**Description:** The novel immunopeptidomics data generated in this study.

**File name:** Supplementary Data 3

**Description:** The source data behind the graphs in the main figures.
