# Supplementary material for: Machine learning reveals limited contribution of trans-only encoded variants to the HLA-DQ immunopeptidome
Source: Commun Biol. 2023 Apr 21;6:442. doi: 10.1038/s42003-023-04749-7 (PMC10121683; doi:10.1038/s42003-023-04749-7)
Supplement: Supplementary file 6 — Reporting Summary [file 42003_2023_4749_MOESM6_ESM.pdf]

nature portfolio

Corresponding author(s): Morten Nielsen

Last updated by author(s): Apr 14, 2023

Reporting Summary

Nature Portfolio wishes to improve the reproducibility of the work that we publish. This form provides structure for consistency and transparency in reporting. For further information on Nature Portfolio policies, see our Editorial Policies and the Editorial Policy Checklist.

Statistics

For all statistical analyses, confirm that the following items are present in the figure legend, table legend, main text, or Methods section.

n/a	Confirmed	
☐	☒	The exact sample size (*n*) for each experimental group/condition, given as a discrete number and unit of measurement
☒	☐	A statement on whether measurements were taken from distinct samples or whether the same sample was measured repeatedly
☐	☒	The statistical test(s) used AND whether they are one- or two-sided<br>*Only common tests should be described solely by name; describe more complex techniques in the Methods section.*
☒	☐	A description of all covariates tested
☒	☐	A description of any assumptions or corrections, such as tests of normality and adjustment for multiple comparisons
☒	☐	A full description of the statistical parameters including central tendency (e.g. means) or other basic estimates (e.g. regression coefficient) AND variation (e.g. standard deviation) or associated estimates of uncertainty (e.g. confidence intervals)
☐	☒	For null hypothesis testing, the test statistic (e.g. *F*, *t*, *r*) with confidence intervals, effect sizes, degrees of freedom and *P* value noted<br>*Give P values as exact values whenever suitable.*
☒	☐	For Bayesian analysis, information on the choice of priors and Markov chain Monte Carlo settings
☒	☐	For hierarchical and complex designs, identification of the appropriate level for tests and full reporting of outcomes
☐	☒	Estimates of effect sizes (e.g. Cohen's *d*, Pearson's *r*), indicating how they were calculated

Our web collection on statistics for biologists contains articles on many of the points above.

Software and code

Policy information about availability of computer code

Data collection	The PEAKS Studio 10.5 software was used to identify the peptide ligand sequences from the LC-MS/MS analysis.
Data analysis	PeakView Software version 1.2.0.3 (AB Sciex, Framingham, MA, USA) was used for spectral analysis and data visualization.<br>Data visualizations in the manuscript figures were made in Python 3.8 using the Matplotlib library (version 3.5.1) and the seaborn library (version 0.12.0). Sequence logos were constructed using Seq2Logo-2.0.

For manuscripts utilizing custom algorithms or software that are central to the research but not yet described in published literature, software must be made available to editors and reviewers. We strongly encourage code deposition in a community repository (e.g. GitHub). See the Nature Portfolio guidelines for submitting code & software for further information.

Data

Policy information about availability of data

All manuscripts must include a data availability statement. This statement should provide the following information, where applicable:

- Accession codes, unique identifiers, or web links for publicly available datasets
- A description of any restrictions on data availability
- For clinical datasets or third party data, please ensure that the statement adheres to our policy

The mass spectrometry proteomics data have been deposited to the ProteomeXchange Consortium via the PRIDE partner repository with the dataset identifier PXD040860 and 10.6019/PXD040860. HLA typing for the 16 BLCLs used in the study are included in Supplementary Data 1. The novel immunopeptidomics data

generated for this study is available in Supplementary Data 2. Numerical source data used to generate the main figures are included in Supplementary Data 3. The training dataset used in the study can be downloaded from the NetMHCIIpan-4.2 web server on the 'Training data sets' page, available at https://services.healthtech.dtu.dk/services/NetMHCIIpan-4.2/.

Human research participants

Policy information about studies involving human research participants and Sex and Gender in Research.

Reporting on sex and gender	Not relevant to our study
Population characteristics	Not relevant to our study
Recruitment	Not relevant to our study
Ethics oversight	Not relevant to our study

Note that full information on the approval of the study protocol must also be provided in the manuscript.

Field-specific reporting

Please select the one below that is the best fit for your research. If you are not sure, read the appropriate sections before making your selection.

☒ Life sciences ☐ Behavioural & social sciences ☐ Ecological, evolutionary & environmental sciences

For a reference copy of the document with all sections, see nature.com/documents/nr-reporting-summary-flat.pdf

Life sciences study design

All studies must disclose on these points even when the disclosure is negative.

Sample size	The training dataset sizes were based on the size of the previously available immunopeptidomics dataset from NetMHCIIpan-4.1, along with the size of the novel HLA-DQ dataset. For performance evaluation, sample sizes in the statistical tests were based on the number of HLA molecules in the training data. For more details refer to Materials and Methods in the manuscript.
Data exclusions	The HLA-DQ immunopeptidomics data was filtered to include only peptides of length 12-21 excluding predicted HLA class I binders. Further, the peptides were then mapped to the human proteome to define peptide context, resulting in around 4% of peptides with no match being discarded.
Replication	The motif analysis was performed on 2 sets of different homozygous BLCLs sharing the same HLA-DQ allele to confirm reproducibility of the motifs obtained for those alleles. (721.221 and IHW09004 shared HLA-DQA1*01:01-DQB1*05:01 allele and IHW09072 and IHW9100 shared HLA-DQA1*04:01-DQB1*04:02 allele)
Randomization	Not relevant for our experimental design (for more details refer to Materials and Methods in the manuscript).
Blinding	Not relevant for our experimental design (for more details refer to Materials and Methods in the manuscript).

Reporting for specific materials, systems and methods

We require information from authors about some types of materials, experimental systems and methods used in many studies. Here, indicate whether each material, system or method listed is relevant to your study. If you are not sure if a list item applies to your research, read the appropriate section before selecting a response.

Materials & experimental systems

n/a	Involved in the study
☐	☒ Antibodies
☐	☒ Eukaryotic cell lines
☒	☐ Palaeontology and archaeology
☒	☐ Animals and other organisms
☒	☐ Clinical data
☒	☐ Dual use research of concern

Methods

n/a	Involved in the study
☒	☐ ChIP-seq
☒	☐ Flow cytometry
☒	☐ MRI-based neuroimaging

Antibodies

Antibodies used	The anti-human HLA-DQ specific monoclonal antibody (clone SPVL3) which recognizes a monomorphic determinant of human class II HLA-DQ molecules was used for affinity purification of total HLA DQ from the BLCLs.
Validation	The anti-human HLA-DQ specific monoclonal antibody (clone SPVL3) is well-characterized and is available at small scale from different commercial sources. The large scale production of this antibody was done in house from the hybridoma cell line (clone SPVL3).

Eukaryotic cell lines

Policy information about cell lines and Sex and Gender in Research

Cell line source(s)	Homozygous B lymphoblastoid cell lines (BLCL) were obtained from the International Histocompatibility Working Group (IHWG) Cell and DNA bank housed at the Fred Hutchinson Cancer Research Center, Seattle, WA (http://www.ihwg.org). A group of 16 cell lines expressing high frequency HLA-DQ alleles were selected for the study.
Authentication	All cell lines were subjected to high-resolution sequencing based HLA typing (HLA-A, -B, -C, DRB1,3, 4, 5, DP and DQ) immediately upon receipt and growth in our laboratory, for authentication prior to large scale culture and data collection. Please also refer to the IHWG for information regarding cell line authentication: https://www.fredhutch.org/en/research/institutes-networks-ircs/international-histocompatibility-working-group.html
Mycoplasma contamination	All cell lines were tested negative for mycoplasma contamination.
Commonly misidentified lines (See ICLAC register)	No commonly misidentified cell line was used in this study.
